# World Trade Center Health Program: First Decade of Research

**DOI:** 10.3390/ijerph17197290

**Published:** 2020-10-06

**Authors:** Albeliz Santiago-Colón, Robert Daniels, Dori Reissman, Kristi Anderson, Geoffrey Calvert, Alexis Caplan, Tania Carreón, Alan Katruska, Travis Kubale, Ruiling Liu, Rhonda Nembhard, W. Allen Robison, James Yiin, John Howard

**Affiliations:** 1 World Trade Center Health Program, National Institute for Occupational Safety and Health (NIOSH), Centers for Disease Control and Prevention (CDC), Washington, DC 20201, USA; ASantiagoColon@cdc.gov (A.S.-C.); dvs7@cdc.gov (D.R.); kga7@cdc.gov (K.A.); jac6@cdc.gov (G.C.); yoy6@cdc.gov (A.C.); tjc5@cdc.gov (T.C.); euc8@cdc.gov (A.K.); tek2@cdc.gov (T.K.); lmq2@cdc.gov (R.L.); ypj4@cdc.gov (R.N.); zkz1@cdc.gov (J.H.); 2 NIOSH Office of Extramural Programs, Atlanta, GA 30329, USA; wer6@cdc.gov (W.A.R.); jcy5@cdc.gov (J.Y.)

**Keywords:** World Trade Center Health Program, 9/11, special populations, emerging medical conditions, disaster epidemiology, review

## Abstract

The terrorist attacks on 11 September 2001 placed nearly a half million people at increased risk of adverse health. Health effects research began shortly after and continues today, now mostly as a coordinated effort under the federally mandated World Trade Center (WTC) Health Program (WTCHP). Established in 2011, the WTCHP provides medical monitoring and treatment of covered health conditions for responders and survivors and maintains a research program aimed to improve the care and well-being of the affected population. By 2020, funds in excess of USD 127 M had been awarded for health effects research. This review describes research findings and provides an overview of the WTCHP and its future directions. The literature was systematically searched for relevant articles published from 11 September 2001 through 30 June 2020. Synthesis was limited to broad categories of mental health, cancer, respiratory disease, vulnerable populations, and emerging conditions. In total, 944 WTC articles were published, including peer-reviewed articles funded by the WTCHP (*n* = 291) and other sources. Research has focused on characterizing the burden and etiology of WTC-related health conditions. As the program moves forward, translational research that directly enhances the care of individuals with chronic mental and physical health conditions is needed.

## 1. Introduction 

On the morning of 11 September 2001, two hijacked commercial airliners were deliberately crashed into towers One and Two of the World Trade Center in lower Manhattan, New York City (NYC). Shortly thereafter, the towers collapsed, resulting in nearly 2800 deaths and thousands injured. Hundreds of thousands of people were exposed to a massive cloud of toxic gases and particulates. Tens of thousands participated in the ensuing rescue, recovery, and clean-up efforts. In total, nearly a half million people are estimated to be at increased risk of adverse health effects from exposures to physical, psychological, and emotional stressors in the days, weeks, and months following the terrorist attacks [1]. 

Shortly after the attacks, federal funds were appropriated to support a variety of post-disaster activities, including the establishment of the World Trade Center Health Registry (Registry). On 2 January 2011, the James Zadroga 9/11 Health and Compensation Act of 2010 (Zadroga Act) was signed into law and the World Trade Center Health Program (WTCHP) was created to monitor and treat responders and survivors with 9/11-related adverse health conditions. The Program is housed under the U.S. Department of Health and Human Services and administered by the Director, National Institute for Occupational Safety and Health (NIOSH). As of 30 June 2020, 105,272 members were enrolled in the WTCHP. 

Part C of the Zadroga Act establishes research of physical and mental health conditions related to 9/11 as a key function of the WTCHP. NIOSH manages the research agenda using its research-to-care framework for prioritizing, conducting, and assessing research primarily intended to inform the clinical care of program members. This report describes the research program now entering its tenth year, with an overview of major findings and future directions.

## 2. World Trade Center Health Program (WTCHP) 

The WTCHP provides medical monitoring and treatment for specific symptoms and health conditions that are presumed to be caused by exposure to the 9/11 attacks on the World Trade Center, the Pentagon, and Shanksville, Pennsylvania. The affected population consists of responders to each attack and WTC survivors. A responder refers to individuals involved in rescue, response, recovery, clean-up and related support activities following the attack. A survivor refers to a resident, building occupant, or worker who was directly impacted and adversely affected by the WTC attacks. The WTCHP also provides for health surveillance involving clinical data collection and analysis and research of health conditions, as described below. Lastly, the WTCHP provides for outreach and maintenance of the Registry, including its research activities.

### 2.1. WTCHP Data Centers and Clinical Centers of Excellence 

Beginning in 2001, federal funds were made available to support specific occupational health clinics throughout the NYC metropolitan area to conduct responder health screening and later grant support for health screening of the affected community members. Over the years, these services grew into periodic medical monitoring and treatment programs for responders that collectively provided health surveillance and patient care for 9/11-related health conditions. These health care services, the Registry, and a nationwide provider network were administratively consolidated under the newly established WTCHP. Five Clinical Centers of Excellence (CCEs) caring for the general responder cohort provide their data to the General Responder Data Center. One CCE caring for responders affiliated with the Fire Department of NYC (FDNY) provide their data to the FDNY Data Center, and one CCE caring for the survivors provide their data to the Survivor Data Center. The CCEs conduct periodic medical monitoring, diagnostic, and treatment services for qualifying conditions, social benefits counseling, and outreach (retention) activities. With respect to medical surveillance, survivors are eligible for periodic monitoring only after diagnosis of a certified condition. Monitoring exams occur every 12–18 months and include a physical examination with a physician with expertise in occupational and environmental medicine, clinical chemistry laboratory tests, spirometry, chest imaging, and updates on self-reported symptoms, diagnoses and quality of life through standardized, cohort-specific physical and mental health questionnaires. Health data that are collected from the CCEs are stored and managed in the three data centers. These data, along with health care claims data, are a primary source of health information made available for research.

Medical screening, monitoring, and treatment services are also conducted by the Nationwide Provider Network (NPN). The NPN is a network of clinics across the country serving eligible responders (NYC, Pentagon, and Shanksville) and survivors (NYC) living outside the New York metropolitan area. Additionally, the William Street Clinic (WSC) provides initial health evaluations and care coordination for some survivors in the New York metropolitan area. The WSC opened in August 2018 to increase capacity for survivor enrollment primarily conducted by the NYC Health + Hospitals CCE. To date, data from WSC and NPN have not been used in WTCHP research.

### 2.2. WTCHP Research Populations

There are three major categories of WTCHP members under study: FDNY responders, general responders, and survivors, which are generally described by the data held in corresponding data centers. FDNY responders are a cohort of active or retired members of the FDNY (e.g., firefighters and emergency medical systems providers) who participated for at least one day in rescue and recovery efforts at any of the former WTC sites (primarily Ground Zero). Yip et al. (2017) provides additional descriptive information on FDNY responders [2]. General responders are rescue, recovery, clean-up, and related support workers or volunteers in the NYC area. The General Responder Cohort is a self-selected group with open enrollment beginning in October 2001 [3]. In general, survivors are people who lived, worked, or went to school, child daycare or adult daycare in the NYC disaster area on 11 September 2001 or in prescribed periods (days weeks, months) after. Eligible times are conditional on survivor exposure potential. There is some overlap between groups. The general responders and NYC survivors are more racially and ethnically diverse compared to FDNY responders who are mostly non-Hispanic, white, and male. Table 1 shows the distribution of WTCHP and Registry member categories. Nearly all were exposed to the WTC attacks. In contrast, Pentagon and Shanksville responders comprise <1% of WTCHP members.

Research is also conducted using self-reported data collected by the Registry. The Registry was initiated in 2002 in collaboration between the New York City Department of Health and Mental Hygiene (NYCDOHMH), Agency for Toxic Substances and Disease Registry (ATSDR), and external scientific and community partners [1]. The Registry was administratively moved to the WTCHP in 2011. It stands as one of the longest running post-disaster registries worldwide. Registrants comprise a diverse group assembled from a combination of potentially exposed residents (adults and children), WTC rescue/recovery workers, students (pre-K through 12), building occupants, and passersby. Eligible participants were enrolled into a closed cohort in 2003–2004 by completing a voluntary baseline health questionnaire (Wave 1). Periodic follow-up surveys were conducted in 2006–2007 (Wave 2), 2011–2012 (Wave 3), and 2015–2016 (Wave 4). A Wave 5 survey is currently underway. Registry study populations are generally described as two mutually exclusive groups of “rescue/recovery workers” and “survivors”. To avoid confusion with WTCHP survivors who meet specific criteria under the Zadroga Act, registrants in the latter group are referred to as community members in this review. Registrants, if eligible, may also be enrolled as members in the WTCHP responder or survivor cohorts.

### 2.3. Environmental Exposure

The WTC complex comprised two 110-floor towers and five other large buildings primarily constructed of reinforced concrete, steel, and glass. Other materials included gypsum, insulation, wallboard, wiring, wood, and office furnishings. The intense fire and collapse of the buildings gave rise to a massive environmental exposure from a dense cloud of suspended pulverized debris and combustion byproducts blanketing much of lower Manhattan. Fires at the WTC site continued intermittently for three months after the attacks [4]. Average airborne levels steeply decreased with time since the attacks and distance from the WTC site. Airborne levels were also influenced by weather patterns. Suspended dust concentrations upwards of 100 mg/m^3^ were estimated to occur in the initial minutes following the collapse [5]. Personal air monitoring among cleanup workers at ground zero in October 2001 resulted in a median dust concentration of 324 µg/m^3^, lowering to 138 µg/m^3^ by April 2002 [4]. Settling dusts resulted in indoor and outdoor contamination that was subject to resuspension. The dusts were complex mixtures of inorganic (e.g., metals, crystalline silica, gypsum, calcite glass fibers, and asbestos) and organic materials (e.g., polycyclic aromatic hydrocarbons, polychlorinated biphenyls, polychlorinated dibenzodioxins, polychlorinated dibenzofurans, and pesticides), including many known toxins and carcinogens [6,7]. Compositions varied by sample location, although dusts were generally alkaline and dominated by nonfibrous debris. Particle size mass concentrations were mostly >53 μm in diameter, with <2% of sampled dusts having an activity median aerodynamic diameter ≤2.5 μm. [4,6,7]. A comprehensive inventory of 9/11 agents is available online from the WTCHP publications webpage [8].

Comprehensive individual level exposure information is not available. Personal and area air monitoring data are sparse. Studies examining exposure–response have used a variety of indirect metrics, alone or in combination, to derive exposure indices, such as arrival time on site, self-reported exposure to the dust cloud, self-reported exposure to dusty home/work settings, and cumulative days worked on the debris pile. As such, exposure indices have varied widely across studies.

## 3. Research Portfolio

### 3.1. Solicitation and Award

**Figure 1:**
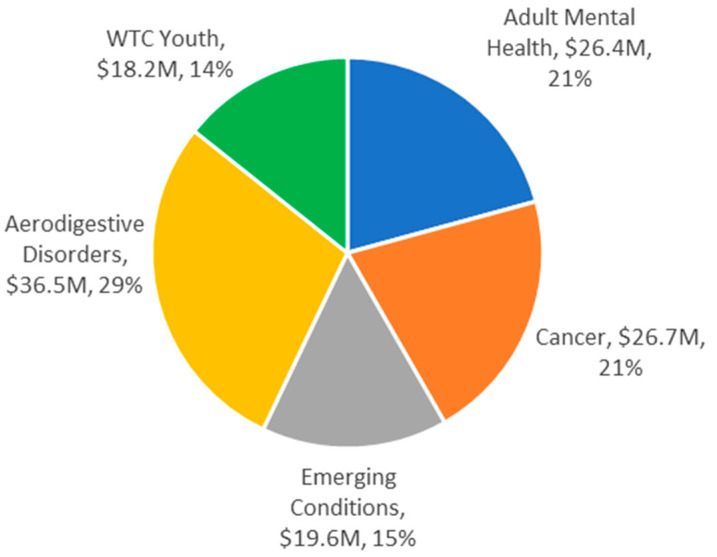
WTCHP Research funding in 2011–2020 by focus area (excluding Registry funding). “WTC Youth” is research targeting health effects among persons exposed before age 18 years and is the largest contributor to the vulnerable population category. (See Section 3.3.4).

Annually the WTCHP solicits applications for scientifically rigorous research in three broad areas listed by the Zadroga Act: (1) research on physical and mental health conditions that may be related to the 11 September 2001, terrorist attacks; (2) research on diagnosing WTC-related health conditions of such individuals, in the case of conditions for which there has been diagnostic uncertainty; and (3) research on treating WTC-related health conditions of such individuals, in the case of conditions for which there has been treatment uncertainty. The solicitation and award processes are managed under the NIOSH Office of Extramural Programs, with peer and program review following the National Institutes of Health (NIH) model for grants and cooperative agreements. From 2011 through June 2020, the WTCHP has funded 84 research projects (75 U01 cooperative agreements, 8 contracts, and 1 R01 research grant), at a cost of nearly USD 128 M (Figure 1 and Table 2). Over the course of the competitive award process, about 28% of U01 applications received have been funded. Among WTC-exposed subpopulations, the highest percentage of applications awarded pertained to research involving responders and persons exposed at ages <18 years (i.e., WTC youth). Most projects awarded pertained to responders, accounting for about 75% of the funds allocated. 

WTCHP also funds research conducted by the Registry. Since 2011, the WTCHP has provided nearly USD 68 M to the Registry, a part of which supported several research projects resulting in over 100 publications.

### 3.2. Stakeholder Involvement in Research Planning

The Zadroga Act established the WTCHP Scientific/Technical Advisory Committee (STAC) and two WTCHP Steering Committees. The STAC comprises scientists, clinicians, and representatives of responders and survivors who review scientific and medical evidence and make recommendations to the WTCHP Administrator on program eligibility criteria, WTC-related health conditions, and research. The STAC was established and operates following the provisions of the Federal Advisory Committee Act. The steering committees include representatives of the affected responder and survivor populations they serve, the CCEs providing services, and pertinent city government offices. The Responders Steering Committee meets to facilitate the coordination of monitoring and treatment programs for the enrolled WTC responders. Similarly, the Survivors Steering Committee provides input for improving health evaluations for screening eligible survivors, and input for the periodic monitoring and treatment programs for certified-eligible WTC survivors. These stakeholder groups meet regularly with WTCHP staff to share important information about ongoing WTCHP activities, including concerns about research findings and future research needs.

### 3.3. Description of WTC Research

The research portfolio was determined by a literature search uncovering relevant research texts published from 2001 through June 2020, including peer-reviewed articles funded by the WTCHP and other entities. Records were included if they were in English and addressed the 9/11 attacks, populations, or pertinent health conditions, care, or outcomes. Synthesis was grouped by broad categories of aerodigestive disorders (Section 3.3.1), adult mental health (Section 3.3.2), cancer (Section 3.3.3), vulnerable populations (Section 3.3.4) and emerging conditions (Section 3.3.5). Articles not classifiable were binned as “other”. 

**Figure 2:**
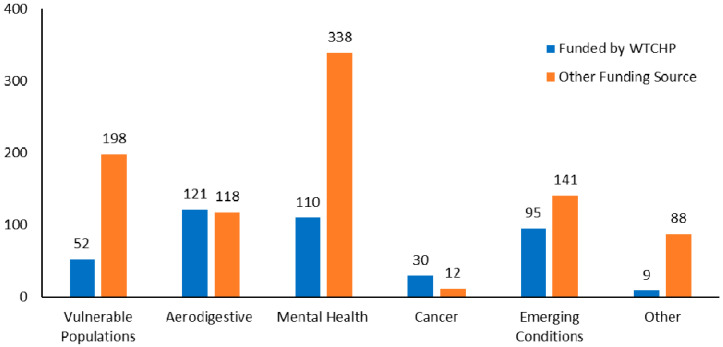
Publication counts by funding source and focus area. Some articles address multiple focus areas (i.e., count sum > 944).

As of 30 June 2020, there were 944 publications eligible for synthesis. Of these, 291 (31%) were funded under the WTCHP. The total literature is nearly equally separated into publications on responders and non-responders (i.e., WTCHP survivors and Registry community members), with several studies including both populations. In contrast, most WTCHP-funded publications (78%) examined responders. In total, about three in four publications examined health burden and etiology, whereas the remainder characterized exposures or interventions (e.g., clinical, health services, and policy research). Among focus areas, most articles (34%) addressed adult mental health conditions, followed by vulnerable populations (19%), aerodigestive disorders (18%), emerging conditions (18%) and cancer (3%). Restricting articles to WTCHP-funded research, the order shifts to aerodigestive disorders (29%), mental health (26%), emerging conditions (23%), vulnerable populations (12%), and cancer (7%) (Figure 2). 

A broad overview of the research is described below. More weight was given to positive findings in efforts to portray the potential health burden.

#### 3.3.1. Aerodigestive Disorders

Approximately 28% of the research dollars competitively awarded was attributed to aerodigestive disorders, which encompassed an array of conditions or diseases of the airway (pharynx and larynx), pulmonary tract (trachea, bronchi, and lungs), and upper digestive tract (esophagus). Aerodigestive disorders comprise the largest WTCHP certification category, accounting for 56% of members with certifications submitted through March 2020. Chronic rhinosinusitis, gastroesophageal reflux disorder (GERD), asthma, sleep apnea, chronic respiratory disorder (from fumes/vapors), and WTC-exacerbated chronic obstructive pulmonary disease are among the top aerodigestive disorders. There is considerable comorbidity within these outcomes, and there is growing evidence linking aerodigestive disorders and posttraumatic stress disorder (PTSD) [9,10,11,12,13,14,15]. Those presenting with multiple conditions generally require more complex treatments and experience poorer outcomes and reduced health-related quality of life [9,13,16]. 

Significant respiratory effects were apparent immediately following the attacks. Within the first 48 h, 90% of rescue workers (*n* = 10,116) reported an acute cough that was regularly accompanied by nasal congestion, chest tightness, or chest burning. Within 6 months, 333 of these responders had a continued WTC-related cough (also known as “World Trade Center cough”) that required four or more consecutive weeks of medical leave. Of these, 173 remained either on medical leave or light duty or were pending a disability retirement evaluation nearly a year later [17]. Spirometry, taken before and after the attacks revealed significant declines in lung function in exposed firefighters shortly following the attacks [18]. This analysis also revealed that persons with predominantly upper-airway symptoms were more likely to return to work compared to those with predominantly lower-airway symptoms. This same study found associations between exposure intensity (measured as time of arrival onsite) and airway hyperreactivity and incidence of World Trade Center cough. Among those presenting with World Trade Center cough, over 80% also reported upper-airway symptoms, such as nasal congestion, nasal drip, and sore throat, and nearly 90% reported symptoms of GERD [18]. 

Overall, studies found higher aerodigestive disorder prevalence among responders than that in the general population and highest in those with greatest exposure [19]. Temporal trends in symptom prevalence have varied, with prevalence of dyspnea, wheeze, rhinosinusitis, and GERD remaining relatively stable over years post 9/11, whereas cough and sore throat have declined steeply within the first 4 years [19,20]. A substantial health burden remains. In a longitudinal study of rescue/recovery workers (*n* = 27,449), the 9-year cumulative incidence was 27.6%, 42.3%, 39.3% for asthma, sinusitis and GERD, respectively [14]. In 2016, the prevalence of age-adjusted asthma and GERD diagnosed after 9/11 in Registry enrollees (*n* = 36,897) was 14.3% and 20.7%, respectively [16]. Similarly, several studies of FDNY responders have shown persistent bronchial hyperreactivity and accelerated declines in lung function in some subjects nearing two decades post-exposure [21,22,23,24].

Similar dose-dependent patterns of lower and upper airway symptoms were observed in the survivor population [25,26]. Within the first year following the attacks, increased respiratory symptoms (predominately a cough, with dyspnea and wheezing to a lesser degree) were observed among residents near the WTC site compared to residents in an unexposed control area. Although they were resolved over time in many exposed residents, symptoms persisted in significant numbers [16,26]. Reasons for response heterogeneity remain elusive, although a recent study of survivors suggested that peripheral airway dysfunction and PTSD may contribute to the persistence of lower respiratory symptoms [27]. 

#### 3.3.2. Mental Health Conditions

A wide variety of mental health disorders fall under the broad category of mental health conditions described in the Zadroga Act, such as PTSD, major and atypical depressive disorders, panic disorder, and various anxiety disorders. Research in this area has been vast, comprising over a fifth (USD 26 M) of the WTCHP research dollars spent. About 22% of certified conditions were for mental health disorders. 

Much of the available research centers on PTSD, a particularly disabling response to traumatic exposure with substantial comorbidity. Extensive literature, beginning immediately following 9/11, has been reviewed in several publications [28,29,30,31,32]. In general, studies suggest a relatively large and persistent exposure-dependent burden of 9/11-related PTSD among the affected population. Injury, loss of loved ones, and witnessed horror are among the strongest predictors [31]. Prevalence rates are heterogeneous across studies, varying widely by study design, time, population, and exposure type (e.g., physical vs. psychosocial). As examples, PTSD prevalence rates within eight weeks of the attacks ranged from 8% to 23% in studies of highly exposed first responders [30] and 8–11% in residents. [33,34]. Less than one-third of studies are longitudinal; however, there is evidence of declining PTSD prevalence over time except for rescue/recovery workers, who appear to have lower prevalence in the first 3 years that increases thereafter [28]. A meta-analysis of 10 studies indicated that responders (e.g., police, firefighters, rescue/recovery workers and volunteers) had lower PTSD risk (odds ratio, OR = 1.61, 95% CI: 1.39, 1.87) compared to civilians (OR = 2.71, 95% CI: 2.35, 3.12) [31]. There were seven experimental or quasi-experimental studies examining PTSD treatments, mostly among residents (*n* = 6), including studies of children (*n* = 2) [35,36,37,38,39,40,41,42]. Treatments, randomization, and study methodologies varied; however, findings generally supported exposure-based intervention therapies to reduce fear-based symptoms [28]. 

There are fewer studies examining major depressive disorder (MDD) in WTC populations. MDD is among the most common illnesses worldwide, contributing over 8% to the global years lived with disability [43]. Although MDD etiology is unclear, there is mounting evidence of increased MDD prevalence from environmental stressors such as mass disasters [44,45,46,47]. A study of the New York metropolitan area residents found a prevalence of 9.4% within 6 months of the WTC attack compared to an expected six-month period prevalence of MDD in unexposed populations of 1.5% to 2.8% [48]. A similar study restricted the sample to Manhattan residents living south of Canal Street (within approximately one mile of the WTC site) found 16.8% with depression-like symptoms [34]. Other surveillance reported symptoms of major depression in 12% of NYC transit workers stationed near the site [49], and 16% of clean up and recovery workers [50]. Although longitudinal data are mostly lacking for this population, there is evidence suggesting many experienced an increase MDD prevalence within six months of the attack, with a subsequent decline to normal baseline rates thereafter [48]. Nevertheless, subgroups exist who are persistently affected or present with recurring symptomatology years after the event [51,52]. This response heterogeneity is consistent with findings from a recent review of 54 studies of trajectories following potential trauma, which suggested four general trajectories, namely (in order by frequency) resilience, recovery, chronic stress, and delayed onset [53].

A recent longitudinal study examining mortality patterns in Registry enrollees compared to the general population found increased deaths from suicide among rescue/recovery workers (SMR = 1.82, 95% CI: 1.35, 2.39), but not community members (SMR = 0.86, 95% CI: 0.53, 1.31) [54]. This is the first and only study reporting increased suicide in a WTC subpopulation. Additional research is needed to clarify this association. Future research should include other investigations aimed to characterize resilience in the affected population, such as studies examining the relationship between mental illness and intentional self-medication. 

#### 3.3.3. Cancer

Cancer accounts for about 13% of WTCHP certified conditions. Non-melanoma skin cancer occurred most often, comprising about 27% of certified cancers, followed by prostate cancer at 21%. Consequentially, cancer research accounts for a large portion (21%) of the funds awarded. There are descriptive and analytic studies examining cancer mortality and incidence, with a few studies examining mechanisms or treatment [54,55,56,57,58,59,60,61,62,63,64,65,66,67,68,69,70,71,72,73,74,75,76]. There are also notable reviews [77,78]. Most information stems from a set of longitudinal studies examining cancer incidence in FDNY responders [55,56], rescue/recovery workers [57,58], and Registry registrants [59,60], which are the focus of this discussion. 

The FDNY study examined cancer patterns in 9853 male firefighters, 8927 of whom were exposed to 9/11 hazardous agents as WTC responders [55]. Follow-up was until 2008. Overall cancer risks were not increased in adjusted analyses compared to the general population (standardized incidence ratio, SIR = 1.02 (95% CI: 0.90, 1.15); however, significantly elevated risk was observed for thyroid cancer (SIR = 2.17, 95% CI: 1.23, 3.82). There was some evidence on modestly increased malignant melanoma, non-Hodgkin lymphoma, and prostate cancer. SIRs were generally increased among exposed firefighters compared to those unexposed. An update was conducted comparing 11,457 FDNY firefighters who were WTC responders to an external control group of 8220 firefighters employed in Chicago, Philadelphia, and San Francisco with follow-up until 2009 [56]. Thyroid cancer remained elevated (relative risk, RR = 3.43, 95% CI: 0.94, 18.94). Prostate cancer was elevated during the latter half of follow-up (2005–2009; RR = 1.38, 95% CI: 1.01, 1.88).

Shapiro et al. (2019) recently updated cancer incidence in 28,729 rescue/recovery workers [58]. Follow-up was through 2013. Cases were identified through linkage with six state tumor registries. Exposure indices were derived from self-reported information on arrival time, exposure to the dust cloud, ever/never working on the debris pile, and cumulative days worked on WTC efforts. Expected numbers of cancer cases were calculated based on state rates and national rates. Exposure-response was examined using multivariable Cox proportional hazards regression for all cancer sites combined and for prostate cancer. SIRs were elevated for all cancer sites combined (SIR = 1.09, 95% CI: 1.02, 1.16), prostate cancer (SIR = 1.25, 95% CI: 1.11, 1.40), thyroid cancer (SIR = 2.19, 95% CI: 1.71, 2.75), and leukemia (SIR = 1.41, 95%: CI: 1.01, 1.92). Regression models did not yield evidence of an exposure–response association for either outcome examined. 

The most recent update on cancer incidence in the Registry cohort included 35,476 community members and 24,863 responders followed through 2011 [60]. Cancers were identified by linkage to 11 state cancer registries; expected numbers of cancers were based on New York State rates. Separate analyses were conducted for responders and community members. Qualitative descriptions of WTC exposures were used to classify exposure as high, intermediate, or low. Prostate cancer and skin melanoma were significantly elevated in both populations (responders: SIR = 1.43, 95% CI: 1.25, 1.63 and SIR = 1.49, 95% CI: 1.05, 2.06, respectively; community members: SIR = 1.27, 95% CI: 1.10, 1.46 and SIR = 1.54, 95% CI: 1.12, 2.07) compared to the general population. Thyroid cancer was significantly elevated only in responders while breast cancer and non-Hodgkin lymphoma were significantly elevated only among community members. Internal analyses showed a significant exposure-response for bladder cancer and log-transformed cumulative exposure scores (HR at unit log-score = 2.18, 95% CI: 1.10, 4.34, *p* = 0.03), but not for any other exposure metric. This finding merits cautious interpretation because log-transformation of the exposure can result in overestimating the response in the low-dose range [79]. A significant exposure-response trend was observed for skin melanoma in community members across ordinal categories of exposure (HR = 1.53, 95% CI: 1.04, 2.23).

Based on these observations, other studies have examined cancers of a priori interest. For example, there is emerging evidence of increased prevalence of monoclonal gammopathy of undetermined significance (MGUS) among WTC-exposed firefighters. MGUS, a nonmalignant outcome, can progress to multiple myeloma in some cases [72]. Another study indicated WTC-dusts are highly capable of inducing ***mdig*** (mineral dust-induced gene, also known as mina53, MINA, or NO52) in normal B cells and malignant myeloma cell lines. The levels of mdig mRNA and protein are associated with multiple myeloma progression and prognosis [74]. Still, the current evidence of increased multiple myeloma in WTC populations is inconsistent. Modestly increased risk was observed in studies of firefighters (SIR = 1.49, 95% CI: 0.56, 3.97; *n* = ≤ 5) and Registry rescue/recovery workers (SIR = 1.35, 95% CI: 0.70, 2.36; *n* = 12); however, confidence intervals were wide [55,60]. An earlier study of Registry rescue/recovery workers followed through until 2008 found significant excess multiple myeloma risk (SIR = 2.85, 95% CI: 1.15, 5.88; *n* = 7) [59]. In contrast, there was no evidence of increased risk in the General Responder cohort (SIR = 0.80, 95% CI: 0.41, 1.40; *n* = 12) or in Registry registrants not involved in rescue/recovery (SIR = 0.67, 95% CI: 0.31, 1.28; *n* = 9) [58,60]. In all studies, case numbers were small, adding to estimate uncertainty. 

Overall, there is evidence of modestly increased cancer risk in the WTC population. The evidence is strongest for all cancers combined and cancer of the thyroid and prostate; however, there were also intermittent indications of other excess cancers, such as bladder cancer, malignant melanoma, multiple myeloma, leukemia, and non-Hodgkin lymphoma. There are notable limitations. First, the observation period is relatively short given complex tumorigenesis that is expected to occur over decades. Continued follow-up is needed to further elucidate cancer risks. Second, errors in within-study comparisons may result from differences in medical monitoring between groups. For example, the observed increased rates of thyroid cancer, a disease with few known risk factors and none seemingly connected to WTC exposure, may be caused by heightened medical surveillance rather than exposure-related disease [73]. Yet others argue against a surveillance bias in thyroid cancer findings [68,69]. Additional research accounting for potential biases is needed. Third, between-study comparisons merit caution given overlap between cohorts. Future studies pooling data from the three cohorts may help address the overlap and increase statistical power [77]. Additional molecular studies focusing on mechanisms may inform etiology, pathophysiology, and ultimately aid clinical management. For example, further exploration of *mdig* expression may result in its use as a prognostic marker to guide multiple myeloma treatments [74].

#### 3.3.4. Vulnerable Populations

Minority groups, pregnant women, women of reproductive age, the elderly, adolescents, and other minor children represent potentially vulnerable classes within the WTC-affected population. Research topics include adverse health effects from prenatal exposures to chemicals and persistent organic pollutants on a variety of health outcomes, longitudinal health and behavioral outcomes, mental health, risk behaviors, health service needs and use, and gene-environment interactions, among others. Research involving persons exposed before age 18 years accounts for about 14% of funds awarded. This group is the major contributor to existing research on vulnerable populations and is the focus of the discussion below. 

##### Prenatal Exposures

Two studies examined gestation and infant growth following WTC prenatal exposure [80,81]. Most studies evaluated health effects (primarily neurodevelopment and cognitive effects) from prenatal exposures to chemicals and persistent organic pollutants. Prenatal chemical exposures included polycyclic aromatic hydrocarbons (PAHs) [81,82,83], perfluoroalkyl substances (PFAS) [84,85,86], mercury [80], and polybrominated diphenyl ethers (PBDEs) [87,88].

Lederman et al. (2004) found infants born to women living within a 2-mile radius of the WTC site within four weeks of the attacks had significantly lower birth weights and shorter birth lengths than term infants born to women living outside the area [89]. These outcomes were only partially mediated by gestation period. In contrast, head circumference, ponderal index, and percentage of small size for gestational age (SGA) births were not associated with distance from the site. They also found that the occurrence of attacks within the first trimester was significantly associated with shortened gestation and slightly smaller head circumference. The head circumference effects were entirely explained by gestational duration. The study sample did not include women exposed during the last trimester; therefore, comparisons were limited to first and second trimesters. Preterm deliveries, stillbirths, and spontaneous abortions were not assessed. It is not clear whether the effects observed were attributable to trauma, environmental exposure, or both. 

Studies measuring benzo[a]pyrene-DNA (BaP-DNA) adducts, a biomarker of PAH exposure, in maternal blood and newborn cord blood found concentrations in the newborns inversely correlated with linear distance (1 mile) from the WTC site [81], and similar to or higher concentrations than in the mothers [82]. Moreover, higher BaP-DNA concentrations in newborns in combination with in utero exposure to environmental tobacco smoke (ETS) were associated with decreased fetal growth [81]. Still, the authors acknowledged that results were limited by modest numbers of participants with adduct measurements and few subjects living or working within the 1-mile radius. A subsequent study continued to follow the children and found that elevated PAH exposure, in combination with ETS exposure, may have contributed to a modest reduction in cognitive development [83]. In addition to limitations of a small sample size, the authors acknowledged that confounding by maternal distress during pregnancy, which has been linked to decreased infant mental development, could not be ruled out. 

Spratlen et al. (2018) found 13% higher perfluorooctanoic acid (PFOA) concentrations in pregnant women living or working within 2 miles of the WTC site compared to the reference group (geometric mean ratio, GMR = 1.13, 95% CI: 1.01, 1.27) [86]. Exposure to several PFAS prenatally have been associated with higher lipid levels and triglycerides in maternal and cord blood [90]. Increasing concentrations of perfluorooctane sulfonate (PFOS) have been associated with higher total lipids (*p*-trend = 0.03) [85,91]. Similarly, PFOA has been associated with higher triglyceride levels, but no association was found for total cholesterol [85,92]. Studies evaluating the relationship between prenatal PFAS exposure and cognition in non-WTC populations have yielded inconsistent findings, showing there is a complex relationship between PFAS and neurodevelopment [93,94,95,96,97,98,99]. A study of 302 mother–child dyads enrolled in a WTC birth cohort found that overall increases in PFAS concentrations were associated with higher Mental Development Index (MDI) scores (higher scores indicating better development), which assesses early cognition and language development by evaluating sensory-perception, knowledge, memory, problem solving and early language [86]. Furthermore, sex-specific relationships between PFAS and cognition were found. Higher cognitive scores (verbal and full-scale IQ) were observed with increases of in PFOS at 2 years old (*p* interaction = 0.04) and increases in PFOA at 4 years old (*p*-interaction = 0.04) for females only. Conversely, principal component analyses found significant associations between principal component 2, comprised of higher perfluorononanoic acid (PFNA) concentrations and lower PFOA and perfluorohexane sulfonic acid (PFHxS) concentrations, and lower psychomotor development index (PDI) scores at age 3 (β = −3.51, 95% CI = −6.01, −1.02) and lower verbal IQ at age 4 (β = −2.67, 95% CI: −5.14, −0.20). 

Prenatal exposure to mercury and PBDEs has been studied in the context of neurodevelopment. Lederman et al., 2008 found an association between higher cord blood mercury and reduced PDI scores at 26 and 48 months old among the offspring of pregnant women who were living or working within 2 miles of the WTC site, after adjusting for fish and seafood consumption during pregnancy [85]. Cowell et al., 2015 detected four PBDE congeners in more than 50% of cord blood samples collected from pregnant women followed for 7 years after giving birth [85,92]. Of seven PBDE congeners evaluated in this study, two were associated with increased attention problems among children at age 4. 

##### Childhood and Adolescence

In addition to evaluating the relationship of prenatal exposures on birth outcomes and early childhood, several studies have assessed the effects of childhood exposures and health effects later in life (i.e., adolescence). 

Among a sample of survivors who were ≤18 years old on 9/11/2001, about a third reported new GERD symptoms on their first clinical visit, in addition to identifying prehypertension or hypertension in almost half of the study population [91]. This study found an association between home dust exposure and reduced high-density lipoprotein (HDL), and elevated triglycerides. Trasande et al., 2017 reported a significant association between higher body mass index (BMI) and PTSD diagnosis (unit change in BMI = 2.06 kg/m^2^, 95% CI: 0.37, 3.74; *p* = 0.02) in linear regression adjusted for sex, race/ethnicity, caloric intake, physical activity, and cotinine concentration [100]. 

The potential effects of PFAS exposure on lung function [90] and cardiometabolic profiles [92] among children aged 0–8 on 11 September 2001 has also been studied. Gaylord et al., 2019 evaluated the relationship between PFAS exposure measured in blood and asthma diagnosis among 118 Registry and 169 comparison adolescents aged 13–22 years at the time of study [90]. All mean PFAS levels were significantly higher in the Registry than in the comparison group. Although some PFAS were associated with increased odds of asthma, these results did not reach statistical significance. In addition, no statistically significant associations were found between serum PFAS and lung function parameters, asthma diagnosis, or eosinophil count. 

PFNA was associated with decreased insulin resistance, and both PFOA and PFNA with increased brachial artery distensibility [92]. Another study of respiratory health and lung function in children under 8 years old exposed to the WTC disaster reported an increased risk of incident asthma compared with a sociodemographically-matched group [101]. Dust cloud (OR = 1.22, 95% CI: 1.10, 1.37) and home dust (OR = 1.12, 95% CI: 1.03, 1.23) exposure were both associated with an increased risk of post-9/11 asthma in analyses controlled for sex, race/ethnicity, BMI category, and tobacco smoke exposure [101]. Household income, unmet healthcare needs, and having at least one mental health condition are factors associated with poor asthma control among adolescents diagnosed with asthma post 9/11 [102]. 

Elevated levels of polychlorinated dibenzo-para-dioxins and polychlorinated dibenzofurans (PCDD/Fs) have been found in Registry adolescents, stemming primarily from byproducts of combustion during the WTC fires [103]. Elevated PCDD/F contamination was found around the WTC site [104,105] and in serum levels of responding firefighters [106]. These chemicals are associated with several adverse health outcomes, such as cancer, diabetes, and impaired reproductive and immunologic function. In a study of Registry adolescents who reported home dust exposure compared to unexposed youths matched on age, sex, race, ethnicity, and income, Kahn et al. (2018) found statistically significantly higher median PCDD/F levels in Registry participants compared to the control group [103]. These elevated levels had persisted for over 12 years since the attacks for 16 of 17 congeners assessed. 

Mental health effects were more widely studied within the WTC youth population. Early studies described the views and reactions of adolescents to the traumatic events of the WTC disaster [107,108,109,110,111]. PTSD, anxiety, and depression are the top three certified mental health conditions among WTCHP youth members. Several studies have evaluated these outcomes [112,113,114,115,116,117,118,119,120]. For example, six months after the WTC attack, New York City students in grades 4–12 with direct WTC exposure reported an increase in media consumption about the WTC attack (e.g., TV, Web, radio, and print media) [121]. The increase in media use was associated with probable PTSD. Among students without direct WTC exposure or family exposure to the WTC attack, intensive media use was also found to be associated with probable PTSD.

Youth groups engaging in risk behaviors, such as substance abuse, and cigarette and alcohol consumption have been reported by some studies [122,123]. Chemtob et al., 2009 reported a five-fold increase in substance abuse in middle and high school students with one WTC exposure risk factor and a 19-fold increase among those with three or more WTC exposure risk factors [122]. In turn, substance abuse was associated with impaired schoolwork, school behavior and grades in that same study. Witnessing a disturbing event on 9/11 and fear for personal safety on 9/11 has been associated with increases in drinking alcohol [123].

As of 30 March 2020, about 5% of Registry enrollees and 3% of currently enrolled WTCHP survivors were aged < 18 years on 11 September 2001. Among the latter group of WTCHP survivors, about 70% were certified for aerodigestive conditions, 46% for mental health conditions, and 13% for cancers. The top three certified conditions were obstructive airway disease (48%), upper respiratory disease (44%) and PTSD (25%). The top four cancers are thyroid cancer (42%), breast cancer (24%), leukemia (24%), and lymphoma (24%). These statistics stem from those survivors who are currently enrolled, which represents only a small fraction of the total population at risk. Estimates of this population have varied widely, ranging from a few thousand to tens of thousands [1,124,125]; therefore, the nature and extent of adverse health effects among those exposed as minors remains largely unknown. Opportunities for future research involving this group and other vulnerable populations are diminishing with time because of natural attrition and other losses, such as relocation outside of the NYC area. Thus, research is urgently needed to better to identify the at-risk populations, characterize the health burden, and inform care. 

#### 3.3.5. Emerging Conditions

Ongoing WTC surveillance and research activities may periodically uncover new health conditions that: (1) appear related to WTC exposures but are not recognized as qualifying conditions for certification and coverage under the WTCHP, or (2) may be medically associated (i.e., due to disease progression or adverse effect of treatment) with a certified WTC-related health condition. In both cases, the emerging condition must be thoroughly evaluated using the best available science to inform the need and extent of program services. Program changes may be necessary, which include, but are not limited to rulemaking to add the condition to the list of covered conditions or changes in existing monitoring and/or treatment. To illustrate potential emerging health conditions, three exploratory research topics are discussed below. 

##### Autoimmune Disease

Systemic autoimmune diseases (SAIDs) are a heterogeneous group of diseases that result in damage to multiple organs stemming from an adverse immune response to self-antigens [126]. Although individually rare, SAIDs substantially impact mortality and morbidity and together are a leading cause of death among women aged < 65 years [127]. Recent studies have suggested associations between SAIDs, PTSD, and WTC-exposure [128,129,130]. A case-control study of responding firefighters found SAIDs incidence increased by 13% (OR = 1.13, 95% CI: 1.02, 1.26) for each additional month worked at the WTC site [130]. Risk were also elevated among those with high acute exposure (working during the morning of 9/11) compared to all others (OR = 1.85, 95% CI: 0.86, 3.89). A subsequent longitudinal study of firefighters did not find significantly different standardized SAID rates among firefighters compared to an external referent (SIR = 0.97; 95% CI: 0.77, 1.21); however, when stratified by exposure, the lower WTC exposure group had 9.9 fewer cases than expected, whereas the higher WTC exposure group had 7.7 excess cases. In evaluating specific diagnoses, systemic lupus erythematosus among firefighters was significantly increased (SIR = 7.33; 95% CI: 3.66, 13.12); however, there were only a few cases (*n* = 11) [129]. Another study reported associations between SAID incidence and intense dust cloud exposure (ever/low-never) in general responders (RR = 1.86; 95% CI: 1.02, 3.40) and between SAIDs and PTSD in community members (RR = 2.8; 95% CI: 1.6, 4.9) [128]. 

In summary, recent studies provide some evidence of increased risk of SAIDs that is linked with increased exposure. There is also emerging evidence of relationships between PTSD, depression, and autoimmune diseases in other populations [131,132,133,134,135]. Yet, disease rarity, complexity, diagnostic imprecision, and poorly understood etiology add to the uncertainty in existing findings. Furthermore, SAIDs comprise a diverse group of diseases that may have differing pathways. More research on specific outcomes grouped by similar pathophysiology is needed. Research is also needed to clarify the roles of mental health conditions, WTC dust exposure, and other factors play in order to elucidate patterns of SAIDs in the WTC population. Future research on mechanisms may inform the causal pathway for SAID and support interventions to improve outcomes.

##### Cardiovascular Disease

Increased risk of cardiovascular disease (CVD) has been linked to airborne particulate exposures [136] and severe psychological stress [136,137,138,139]; therefore, WTC exposures and experiences may potentially increase CVD risks. The relationship between WTC exposures and CVD has been examined in several studies [54,76,140,141,142,143,144,145,146,147]. Registry enrollees were studied most often [54,141,143,144,145,146,147]. In a study of community members (*n* = 8418) from this population, early surveillance revealed increased odds of self-reported stroke among those exposed to the dust cloud compared to unexposed enrollees (OR = 5.6, 95% CI: 1.3, 24.4) [143]. Another study reported Registry enrollees (*n* = 42,527) with PTSD had increased risk of self-reported stroke (HR = 1.64, 95% CI: 1.37, 1.96), as did those with intense dust exposure (HR = 1.20, 95% CI: 1.02, 1.42) [147]. In a prospective study of 39,234 Registry participants, self-reported heart disease (defined as physician-diagnosed angina, heart attack, and/or other heart disease) was significantly associated with intense dust cloud exposure in women (HR = 1.28, 95% CI: 1.02, 1.61) but not men (HR = 1.14, 95% CI: 0.97, 1.34). PTSD at enrollment was significant in women (HR = 1.68, 95% CI: 1.33, 2.12) and men (HR = 1.62, 95% CI: 1.34, 1.96) [141]. Point estimates were diminished in models jointly examining PTSD and dust cloud exposure (as independent covariates); however, PTSD estimates remained significant in men and women. There was evidence in multiple studies of an exposure–response association between heart disease and acute injuries sustained during the attacks [141,144,146] in the Registry population. Most Registry studies rely on self-reported data; however, a longitudinal study used outcome data obtained by linkage to a New York State hospital discharge-reporting system. That study found elevated heart disease hospitalization among women (HR = 1.32, 95% CI: 1.01, 1.71), but not men, with self-reported PTSD at enrollment. There was also an association between rescue/recovery-related exposure and heart disease hospitalization in men (HR = 1.82, 95% CI: 1.06, 3.13) comparing high- vs. low-exposure categories. Excess risk was also observed in women, although the estimate was not statistically significant (HR = 3.29, 95% CI: 0.89, 12.69) [145]. 

In a recent cohort study of responding male firefighters (*n* = 9796), age-adjusted incident rates of CVD (defined as myocardial infarction, stroke, unstable angina, coronary artery surgery or angioplasty, or CVD death from electronic medical records) were higher among firefighters with greater WTC exposure (HR = 1.44, 95% Cl: 1.09, 1.90), while controlling for other risk factors [140]. Excess risk of myocardial infarction (HR = 2.22, 95% CI: 1.30–3.82) and stroke (HR = 2.51, 95% CI: 1.39, 4.47) was evident in a prospective study of general responders with PTSD compared to those without, in multivariable models adjusting for age, gender, body mass index (BMI), blood pressure, smoking, respirator use, and total cholesterol [142]. However, this study provided no evidence of an association between WTC dust exposure and CVD. 

Findings among these studies are inconsistent. Some studies reported significant associations between markers of WTC dust cloud exposure (e.g., self-report of dust cloud exposure, arrival time on site, work on the pile, duration on site) and CVD outcomes [140,141,143,144,145,147], while others have not [54,142,146]. Most studies assessed CVD outcomes as a group including conditions that may not share the same pathophysiology and risk factors (e.g., hemorrhagic stroke). Some outcome definitions were not fully described; therefore, it is difficult to interpret study findings and compare findings across studies [141,144]. These inconsistencies add to the uncertainty in the existing evidence. Other limitations include a general reliance on self-reported data in Registry studies and exposure misclassification common to all WTC studies. Further research is needed to address study limitations, replicate findings, and clarify the health burden from CVD. In addition, research is needed to elucidate mechanisms of causal relationships between WTC-exposure and CVD in the presence of other risk factors, either independently or through mediation by PTSD. 

##### Cognitive Impairment

There is growing evidence of early onset cognitive impairment in the WTC-exposed population [148,149,150,151]. Two causal pathways have been suggested for exposure-related disease. First, recurrent symptoms of PTSD or MDD among those who experienced the attacks may lead to cognitive impairment. Previous studies of veterans have found evidence of an association between PTSD and decreased cognition [152,153], with recent studies of WTC responders showing similar results [149,151]. For example, Clouston et al. (2016) examined a sample of WTC responders (*n* = 813), with an average age of 53 years, and found one in seven screened positive for cognitive impairment [151]. Current PTSD or MDD were associated with a two-fold increase in cognitive impairment. These findings were confirmed in a subsequent study, which also provided evidence of poorer cognitive functioning among WTC responders compared to general population norms [149]. Similarly, a large longitudinal study of a sample of WTC responders (*n* = 1800) found an association between PTSD symptom severity and increased risk of cognitive impairment (hazard ratio, HR = 2.67, 95% CI: 1.33, 5.37) [154]. There are some results suggesting neurodegenerative effects. For example, reductions in gray matter volumes, including in the amygdala and hippocampus, were observed in individuals residing near the WTC [155]. Researchers have also identified associations between PTSD and a variety of physical functional limitations including reductions in walking speed, chair-rise speed, and balance problems [156]. Additional research demonstrated reduced maximal hand grip strength in WTC responders with PTSD and MDD [157]. Investigating neurodegenerative mechanisms, a recent pilot study (*n* = 34) examined plasma-based neuropathological biomarkers in WTC responders and found an association between PTSD and reduced plasma amyloid-β (Aβ) and increased Aβ42/Aβ40 ratios suggesting a neurodegenerative pathway [150]. This was further supported by a follow-up study of a much larger sample of WTC responders at midlife (*n* = 398), which showed that amyloid-1–42 and total tau distributions were associated with increased risk of cognitive impairment in this population [71]. Finally, recent work examining gene expression among responders with PTSD first identified (*n* = 324) differential expression of two genes (NDUFA1, CCDC85B) previously found to be dysregulated in neurodegenerative disorders [158] and, when interrogating gene expression in cell subpopulations among responders (*n* = 39) with/without PTSD, also identified PI4KAP1, REST, and SEPT4 genes in monocyte populations that have been previously implicated in neuronal loss and neurogenesis [159].

There is mounting evidence from human and animal studies indicating that inhalation of air pollutants can increase the risk of neurodegenerative diseases, including cognitively impairing diseases [160,161,162,163,164,165,166,167]. Exposure–response associations between proxies of WTC dust exposure and measures of cognitive impairment have been observed [148]. For example, WTC first responders with high-intensity exposures were more likely to report subjective cognitive deficits compared to responders with lower-level WTC exposure [148]. In another study of responders, the authors found an association between increased time working on-site and lower cognitive functioning [149]. In a longitudinal study the responder analyses confirmed this result among responders carrying the apolipoprotein-4 genetic risk allele (HR = 3.89, 95% 1.79, 8.46) that is believed to cause blood–brain barrier dysregulation [154]. Still, there is far less evidence supporting this pathway and it is difficult to disentangle the effects from environmental exposures and PTSD. 

The at-risk population is entering a time when aging is becoming more clinically significant. Emerging evidence suggests a relationship between WTC-exposure and cognitive impairment risk. Although prognosis is unknown, some mild cognitive impairment cases may progress into seriously debilitating disease. Thus, continued surveillance and research are necessary to strengthen the evidence, better characterize the potential health burden, and inform risk management strategies. In particular, mechanisms of WTC cognitive impairment, either by PTSD/MDD, dust exposure, or both, are largely unknown; therefore, research is needed to clarify the cause and effect, which in turn may lead to more effective interventions and improvements in patient care.

## 4. Future Directions

In December 2015, provisions of the Zadroga Act were extended to 2090, including continued research into health conditions in the affected population. To date, most research has focused on characterizing the public health burden resulting from the 9/11 attacks and improving the understanding of the etiology of WTC health conditions. Still, many unanswered questions on covered conditions remain and there are a number of emerging conditions that need to be addressed. Thus, sustained medical surveillance and etiologic research is an important part of future research until all health risks are sufficiently elucidated. For example, future molecular and biomarker research may reveal new knowledge on disease mechanisms that could lead to more effective treatment strategies for patients with a currently poor prognosis. 

As the program moves forward, more emphasis must be placed on translational research that is aimed to improve care of individuals with chronic mental and physical health conditions. The research will include examinations of treatments, health services, and policies to determine the extent to which patients with WTC-related health conditions are receiving adequate treatment or are responsive to care. Future research will examine knowledge gaps, especially in areas of causal determinates, disease progression, and emerging health conditions. Research targeting potentially understudied outcomes (e.g., reproductive and developmental endpoints) and population subgroups (e.g., minorities, and individuals exposed as children) may add to the knowledge base and help clarify the generalizability of existing findings. Finally, research is needed to inform prevention/mitigation strategies intended to reduce adverse health effects in populations affected by future disasters. This research should include a synthesis of the impact on the policies and practices of emergency management stemming from the 9/11 attacks.

## 5. Conclusions

The tragic events of 9/11 have placed nearly a half million individuals at increased risk of a wide variety of adverse physical and mental health conditions. Health research strategies led by local government and private institutions began almost immediately after the attacks and continues today as a federally-funded and coordinated health research program. This research has led to an extensive literature on WTC-related health effects, which is essential to characterizing the health burden and promoting effective treatment. Nearing the program’s tenth anniversary, the WTCHP strives to address continuing and emerging WTC-health conditions in this large population through research solicitation and assessment. In doing so, NIOSH continues to seek insight from scientists, community members, patients, and other stakeholders in developing an impactful research agenda. 

## Figures and Tables

**Table 1:** World Trade Center Health Program (WTCHP) and Registry Members *.

Characteristic	FDNY Responder Cohort	General Responder Cohort	NYC Survivor Cohort	Registry (as of 4 August 2020)
Number enrolled	15,328	43,811	13,569	65,717
Percent deceased	3.2	2.9	2.1	8.0 ^†^
Percent male	97	86	50	60
Percent Caucasian	87	78	40	63
Mean age on 9/11 (years)	39.8	38.6	42.2	39.1
Percent aged 65+	20	17	53	31

* Restricted to WTC-exposed as of 31 December 2019 unless otherwise indicated. Data from WSC and NPN are excluded. Data were abstracted from data center annual surveillance reports and from Registry Wave 1 data, excluding 5709 registrants who were deceased or withdrawn. ^†^ The percentage of deceased and withdrawn registrants is shown. Abbreviations: FDNY, fire department of the city of New York; NPN, National Provider Network; NYC, New York City, Registry, World Trade Center Health Registry; WSC, William Street Clinic.

**Table 2:** WTCHP research funding from 2011 through June 2020 by group of interest *.

Subpopulation	Solicitation and Award (U01 Only)		Funding (millions)		
	No. Applications	No. Funded (%) ^†^	Awarded Amount	Other Source ^‡^	Total (%)
Responders only	183	56 (30.6)	83.6	10.0	93.6 (73.5)
Responders & survivors	19	2 (10.5)	1.5	0	1.5 (1.2)
Survivors excl. WTC youth	23	4 (17.4)	5.4	1.5	6.9 (5.4)
WTC youth only	28	9 (32.1)	18.2	0	18.2 (14.3)
Other ^§^	13	4 (30.8)	4.5	2.7	7.2 (5.7)
Totals:	266	75 (28.2)	113.2	14.2	127.4

* Excludes Registry funding. ^†^ Excludes one study of responders awarded in 2020 but funding was deferred until 2021. ^‡^ WTCHP funding under contracts (*n* = 8) and other solicitation (*n* = 1 Research Project (R01) grant). ^§^ The group under study was not WTC-exposed (e.g., animal studies, intergenerational studies). Abbreviations: U01, Research Project Cooperative Agreement; WTC, World Trade Center.
